# Leveraging GWAS-Identified Markers in Combination with Bayesian and Machine Learning Models to Improve Genomic Selection in Soybean

**DOI:** 10.3390/ijms26199586

**Published:** 2025-10-01

**Authors:** Yongguo Xue, Xiaofei Tang, Xiaoyue Zhu, Ruixin Zhang, Yubo Yao, Dan Cao, Wenjin He, Qi Liu, Xiaoyan Luan, Yongjun Shu, Xinlei Liu

**Affiliations:** 1 Soybean Research Institute, Heilongjiang Academy of Agricultural Sciences, Harbin 150086, China; xyg81@126.com (Y.X.); xftang@126.com (X.T.); yaoyubo2009@aliyun.com (Y.Y.); caodan825@163.com (D.C.); nkydds@126.com (W.H.); liuqi0316@163.com (Q.L.); luanxiaoyan1201@163.com (X.L.); 2 Key Laboratory of Molecular Cytogenetics and Genetic Breeding of Heilongjiang Province, College of Life Science and Technology, Harbin Normal University, Harbin 150025, China; zhuxiaoyue2001@126.com (X.Z.); zrxin2001@126.com (R.Z.)

**Keywords:** soybean, GWAS, GS, machine learning, SNP

## Abstract

Soybean (*Glycine max* (L.) Merr.) is one of the most important global economic crops, extensively utilized in the production of food, animal feed, and industrial raw materials. As the demand for soybeans continues to rise, improving both the yield and quality of soybeans has become a central focus of agricultural research. To accelerate the genetic improvement of soybean, genome selection (GS) and genome-wide association studies (GWAS) have emerged as effective tools and have been widely applied in various crops. In this study, we conducted GWAS and GS model evaluations across five soybean phenotypes (Glycitin content, Oil, Pod, Total isoflavone content, and Total tocopherol content) to explore the effectiveness of different GWAS methods and GS models in soybean genetic improvement. We applied several GWAS methods, including fastGWA, BOLT-LMM, FarmCPU, GLM, and MLM, and compared the predictive performance of various GS models, such as BayesA, BayesB, BayesC, BL, BRR, SVR_poly, SVR_linear, Ridge, PLS_Regression, and Linear_Regression. Our results indicate that markers selected through GWAS, when used in GS, achieved a prediction accuracy of 0.94 at a 5 K density. Furthermore, Bayesian models proved to be more stable than machine learning models. Overall, this study offers new insights into soybean genome selection and provides a scientific foundation for future soybean breeding strategies.

## 1. Introduction

Soybean (*Glycine max* (L.) Merr.) is one of the most important global food and oilseed crops, widely utilized in various sectors including food, animal feed, and industrial raw materials [1]. As a rich source of protein and oil, soybean plays a critical role in global agricultural production. With the growth of the global population and the evolving demands for food, the need for soybean improvement has become increasingly urgent [2]. In recent years, soybean breeding objectives have expanded to include multiple traits, such as yield, oil content, isoflavone content, and stress tolerance. This study focuses on five soybean traits of important agricultural and nutritional value: glycine content, oil content, pod number, total isoflavone content, and total tocopherol content. These traits not only directly impact the economic value of soybeans but are also closely linked to their nutritional quality. For instance, glycine, a bioactive amino acid, plays a crucial role in the nutritional value of soybeans and is also associated with antioxidant and anticancer health benefits. Oil content, a key determinant of soybean’s economic value, is an important nutritional component rich in unsaturated fatty acids. Pod number is a critical trait influencing soybean yield and is vital for yield improvement. Furthermore, soybean isoflavones, a class of phytoestrogens, and tocopherols, the primary form of vitamin E, are two essential bioactive compounds in soybeans with important health benefits. Soybean isoflavones are recognized for their antioxidant, anticancer, and cardiovascular protective effects, widely utilized in health supplements. Tocopherols, as lipophilic antioxidants, effectively neutralize free radicals, reducing oxidative damage and protecting cells from aging and disease. Among the tocopherols, γ-tocopherol (γ-T) is the most abundant, accounting for approximately 70% of the total, followed by δ-tocopherol (δ-T) and α-tocopherol (α-T). These traits not only affect the market value of soybeans but are also integral to their nutritional composition [3]. As modern breeding objectives diversify, enhancing the selection efficiency and accuracy of these complex traits has become a critical challenge in soybean breeding.

Genome-wide association studies (GWAS) have become an essential tool in plant genetics, providing a genomic perspective for trait improvement by revealing the associations between genotype and phenotype. GWAS enables the identification of SNP (single nucleotide polymorphism) markers across the entire genome that are associated with target traits, thus uncovering the genetic basis of these traits [4]. In soybean, GWAS has been widely applied to explore genes related to important traits such as yield, oil content, and stress tolerance. For instance, in 2022, Zongbiao Duan et al. [5] identified that the natural allele variation of GmST05 primarily controls seed thickness and size in soybean germplasm through GWAS analysis. Similarly, Chao Qin et al. [6] in 2023 identified a gene on chromosome 13, PH13, that regulates plant height and encodes a WD40 protein, with three main haplotypes present in natural populations. Overall, GWAS studies have led to the identification of several candidate genes and key QTLs (quantitative trait loci), providing valuable genetic resources for breeding. However, due to the complexity of the soybean genome and the polygenic nature of traits, GWAS results are often influenced by factors such as population structure and genetic similarity. As a result, more efficient and precise methods are required to leverage these markers effectively [7]. With the advent of the information age, GWAS models have evolved. Early GWAS studies primarily relied on generalized linear models (GLM), a basic statistical method used to analyze the association between genotype and phenotype data [8]. However, a major limitation of GLM is its failure to account for population structure and relatedness, which can lead to false positives in studies involving large populations and complex traits. To overcome these limitations and address challenges such as high computational costs due to large data volumes, algorithms like MLM, LMM, and FarmCPU have been developed [9,10,11,12]. With the continuous advancement and refinement of GWAS methods and models, soybean breeders and researchers are now better equipped to decode the genetic basis of soybean traits, optimize breeding strategies, and accelerate the selection of desirable traits. This progress is crucial for the identification of superior parents, guiding variety improvement, enhancing genetic diversity in the soybean gene pool, and advancing research into stress tolerance and adaptability [13,14,15].

Genome selection (GS), as an emerging genetic improvement technique, provides an effective means to enhance the accuracy of selecting complex traits. By integrating whole-genome markers with phenotypic data, GS uses computational models to predict an individual’s genetic potential, enabling the early selection of individuals with desirable traits [15]. Compared to traditional phenotypic selection methods, GS accelerates the breeding process by predicting trait performance using genotype information, even in the absence of clear phenotypic data. This is particularly advantageous in the improvement of polygenic traits. By leveraging the genetic information from the entire genome, GS can simultaneously optimize multiple target traits, improve prediction accuracy, and reduce the cost and time associated with the breeding process [16]. Moreover, GS effectively addresses the limitations of traditional breeding methods when dealing with complex traits [17]. In recent years, with advancements in computational technology and continuous improvements in algorithms, the application potential of genome selection in plant breeding has gained broader recognition, making it one of the key technologies for enhancing breeding efficiency and accuracy.

In recent years, the combined application of GWAS and GS has gained increasing attention in crop genetic improvement. By integrating the candidate SNPs identified through GWAS with genome selection models, this approach combines phenotypic information with whole-genome marker data, thereby enhancing the accuracy of trait prediction and the precision of genetic effect estimation. GWAS reveals the initial associations between genotype and phenotype, identifying potential candidate genes or SNPs, while genome selection utilizes these markers and broader genetic information to make precise predictions for complex traits [18]. The combination of these two approaches not only makes the breeding process more efficient but also allows for the simultaneous optimization of multiple complex traits. In plant breeding, the strategy of combining GWAS with genome selection has been shown to improve breeding efficiency [17]. In a 2024 study, a marker set composed of the top 1000 SNPs identified through GWAS showed higher prediction accuracy for four traits related to plant height (PH), with accuracy ranging from 0.94 to 0.97 [19]. Another study on genomic selection for Root System Architecture (RSA) in alfalfa found that when all markers were used for prediction, the accuracy was only between 0.11 and 0.18. However, when phenotype-related markers were incorporated, prediction accuracy increased to between 0.7 and 0.8, an improvement of nearly eightfold [20]. These findings provide novel strategies for plant breeding.

In this study, we aimed to investigate the impact of integrating GWAS and genome selection (GS) on improving prediction accuracy. We systematically combined and compared different GWAS and GS models to evaluate their effectiveness. The goal of this research is to combine GWAS with genome selection to enhance the prediction accuracy and improvement efficiency of multiple important traits in soybean. We performed GWAS analysis using five different models to identify key SNP markers associated with traits such as Glycitin content, Oil, Pod, Total isoflavone content, and Total tocopherol content. These results were then integrated with ten Bayesian and machine learning models to optimize the prediction models for genome selection. This comprehensive approach provides more precise and efficient technical support for soybean precision breeding, further driving innovation and development in soybean breeding technologies.

## 2. Results

### 2.1. Phenotypic Analysis

**Figure 1:**
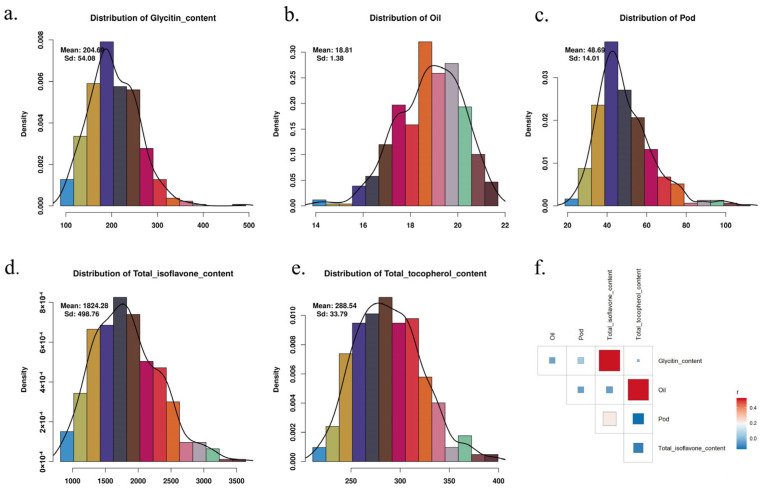
Distribution of Five Soybean Phenotypes and Correlation Matrix. (**a**) Distribution of Glycitin content, (**b**) distribution of Oil, (**c**) distribution of Pod, (**d**) distribution of Total isoflavone content, and (**e**) distribution of Total tocopherol content across soybean samples. The mean and standard deviation for each trait are displayed on the respective plots. (**f**) Correlation matrix showing the relationships between the five traits, with important positive correlations observed between Glycitin content and Total isoflavone content, as well as between Oil and Total tocopherol content. Weak correlations are noted between Oil and Pod, as well as other phenotypes.

To examine the distribution characteristics of five phenotypes in soybean samples, descriptive statistical analysis was first performed on Glycitin content, Oil, Pod, Total isoflavone content, and Total tocopherol content, and corresponding distribution plots were generated (Figure 1). The mean values and standard deviations for each phenotype were also calculated and labeled in the figure. The distribution of Glycitin content exhibited a left-skewed pattern, with a mean of 204.69 and a standard deviation of 54.08 (Figure 1a). This indicates that the majority of soybean samples had lower Glycitin content, but some samples with higher content caused the distribution to be skewed. The distribution of Oil was relatively symmetrical, with a mean of 18.81 and a standard deviation of 1.38 (Figure 1b), showing a typical normal distribution where the oil content in most soybeans is concentrated around 18, with minimal variation among samples. The distribution of Pod showed a broader range, with a mean of 48.69 and a standard deviation of 14.01 (Figure 1c). The Total isoflavone content phenotype had a mean of 1824.28 and a standard deviation of 498.76 (Figure 1d). Most samples had isoflavone content between 1500 and 2500, though some samples exhibited higher values, possibly due to genetic background or environmental factors. The distribution of Total tocopherol content was relatively symmetric, with a mean of 288.54 and a standard deviation of 33.79 (Figure 1e). The distribution was concentrated, with most samples’ tocopherol content falling between 275 and 320, indicating consistent levels of tocopherol across the samples.

To further investigate the relationships between these phenotypes, Figure 1f presents the correlation matrix for the five phenotypes. The results indicate important positive correlations between Glycitin content and Total isoflavone content, as well as between Oil and Total tocopherol content (r ≈ 0.4). However, the correlation between Oil and Pod, as well as with other phenotypes, was relatively weak. These findings provide a foundation for subsequent GWAS analyses, particularly in understanding the potential relationships between phenotypes and the influence of multiple genetic factors.

### 2.2. Assessing the Heritability of Traits

To assess the heritability of each phenotype, we used the REML method in GCTA software(Version 1.95.0) to estimate heritability and calculate genetic variance (V_g_) and environmental variance (V_e_) for the five phenotypes. The results are summarized in Table 1, where heritability (h^2^) reflects the proportion of phenotypic variation attributable to genetic factors. For Glycitin content, the heritability was 0.583, with a *p*-value of 2.70 × 10^−12^, indicating important genetic influence on this phenotype (Table 1). The genetic variance (V_g_) was 1705.95 ± 243.97, and the environmental variance (V_e_) was 1218.31 ± 125.79, showing a substantial contribution of genetic factors to this phenotype. For Oil, the heritability was 0.628, with a *p*-value of 8.43 × 10^−20^, suggesting that genetic factors dominate the variation in oil content (Table 1). The genetic variance (V_g_) was 1.1883 ± 0.1305, and the environmental variance (V_e_) was 0.7046 ± 0.0668, further confirming the important genetic contribution to this trait. For Pod, the heritability was low at 0.174, with a *p*-value of 0.00136. Although genetic influence was important, the low heritability indicates that environmental factors play a major role in the variation of pod number. The genetic variance (V_g_) was 34.058 ± 10.637, and the environmental variance (V_e_) was 162.193 ± 15.321, revealing a large disparity between genetic and environmental variances, suggesting that this phenotype is influenced by multiple factors. For Total isoflavone content, the heritability was 0.466, with a *p*-value of 9.97 × 10^−9^. The genetic variance (V_g_) was 115,903 ± 20,222.9, and the environmental variance (V_e_) was 132,855 ± 13,745.6. The moderate heritability indicates that both genetic and environmental factors contribute to the variation in this trait. For Total tocopherol content, the heritability was 0.586, with a *p*-value of 7.36 × 10^−14^. The genetic variance (V_g_) was 668.987 ± 89.422, and the environmental variance (V_e_) was 472.818 ± 45.603, showing that genetic factors have a substantial effect on this phenotype. Overall, Oil and Glycitin content exhibited higher heritability, indicating that the variation in these traits is predominantly governed by genetic factors. In contrast, Pod had a lower heritability, emphasizing the greater influence of environmental factors on this trait’s variation. These heritability estimates provide important insights for subsequent genome selection, helping to identify phenotypes with a strong genetic foundation.

### 2.3. GWAS Analysis

**Figure 2:**
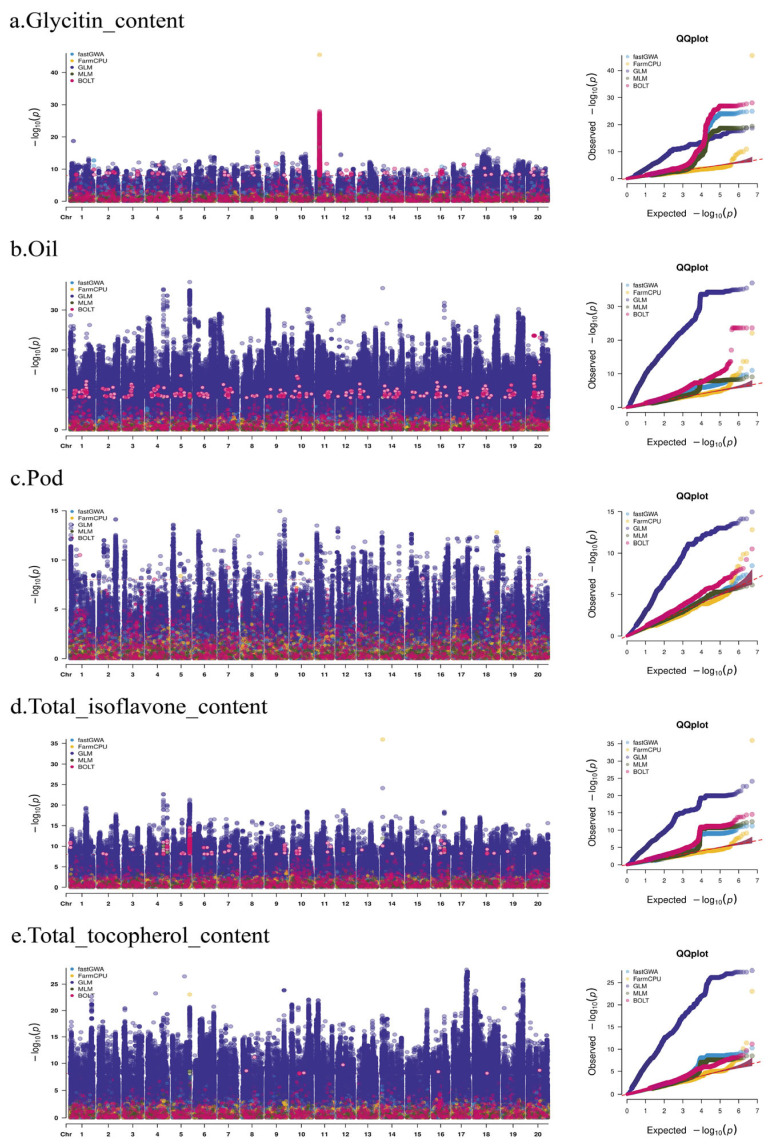
GWAS Analysis of Five Soybean Phenotypes Using Different Methods. Each panel presents the Manhattan plot and QQ plot for the respective phenotype, showing the results from fastGWA, FarmCPU, GLM, MLM, and BOLT methods. The Manhattan plots illustrate the significance of SNPs across the genome, with the *y*-axis representing the negative logarithm of the *p*-value (−log_10_(P)). The QQ plots compare the observed and expected *p*-values, revealing the fit of each method. Important peaks are highlighted in various colors to indicate SNPs detected by each method.

The results of the GWAS analysis for the five phenotypes using different methods, including fastGWA, FarmCPU, GLM, MLM, and BOLT, are shown in Figure 2. For each phenotype (a. Glycitin content, b. Oil, c. Pod, d. Total isoflavone content, e. Total tocopherol content), Manhattan plots and QQ plots demonstrate the performance of different methods in SNP identification. For the Glycitin content phenotype (Figure 2a), BOLT detected important SNP markers, particularly on chromosome 11, where a prominent peak was observed. FarmCPU also identified an important SNP at the same location as BOLT. fastGWA, GLM, and MLM detected multiple SNP markers, although their *p*-values were not as low as those identified by fastGWA. In the analysis of other traits, the GLM model detected the most markers, but it also had the highest false-positive rate. FarmCPU detected important markers at certain loci, though these were typically isolated points. Overall, BOLT and FarmCPU demonstrated more stable performance.

**Figure 3:**
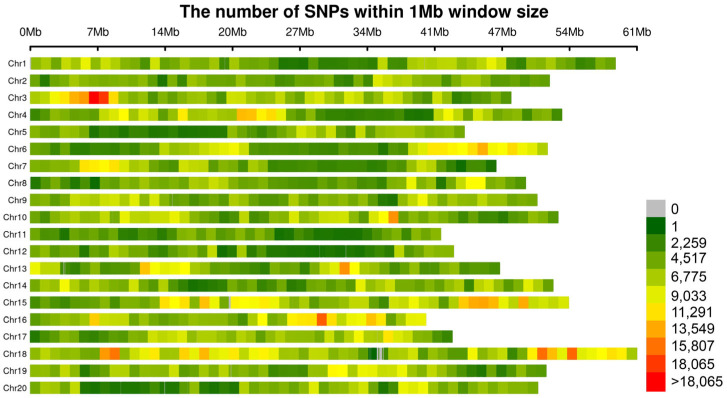
SNP Distribution within 1 Mb Windows Across Soybean Chromosomes. This figure displays the distribution of SNPs within a 1 Mb window across the chromosomes of soybean. The colors represent the SNP count within each window, with the color gradient ranging from light green (indicating 0 SNPs) to red (indicating >18,065 SNPs). The exact values of SNPs are represented by the color scale on the right side of the figure.

Figure 3 shows the distribution of SNPs within a 1 Mb window. There was considerable variation in SNP density across different chromosomes, with some chromosomes (such as Chr3 and Chr18) showing higher SNP density, while others had relatively fewer SNPs. This finding did not fully align with the distribution of important loci in the Manhattan plots, which may be due to the presence of multiple relatively independent effect regions in the genome, the varying genetic associations with phenotypes across chromosomes, and the combined influence of genetic and environmental factors. Additionally, we recorded the computation time for each GWAS model to assess its computational efficiency. As shown in Table 2, the running time for the BOLT and fastGWA models was importantly shorter compared to other models, indicating that these two models are more suitable for large datasets.

### 2.4. SNP Effect Size and Allele Frequency Distribution

**Figure 4:**
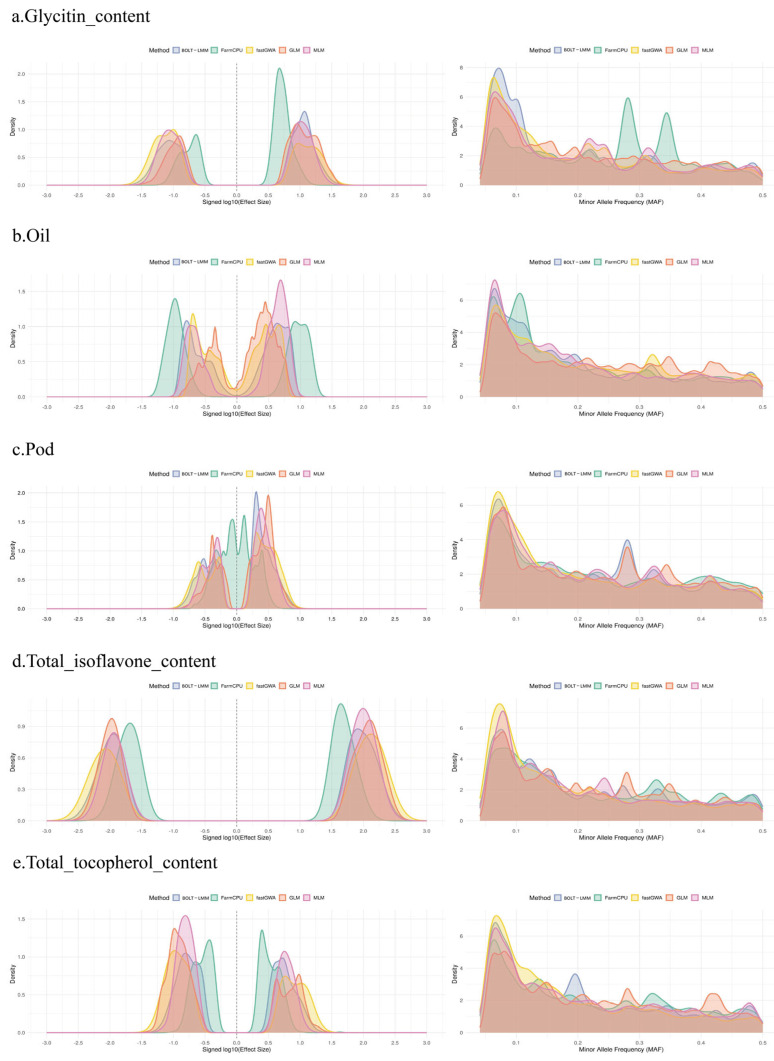
Distribution of SNP Effect Sizes and Minor Allele Frequencies Across Five Soybean Phenotypes. The left panels show the distribution of SNP effect sizes (Signed log10 of effect size), while the right panels display the distribution of minor allele frequencies (MAF). The results are presented for five different GWAS methods: BOLT-LMM (blue), FarmCPU (green), fastGWA (yellow), GLM (orange), and MLM (pink). The plots reveal the differences in SNP effect size and MAF distribution for each phenotype, with varying performance across methods.

To further investigate the relationship between the genetic effect size of SNPs and the minor allele frequency (MAF) for each phenotype, we analyzed the results generated by five different GWAS methods (BOLT-LMM, FarmCPU, fastGWA, GLM, and MLM). Figure 4 illustrates the distribution of SNP effect sizes and MAF for each phenotype, revealing the differences in SNP effect detection and minor allele frequency across the methods. The results indicate important differences in the ability of various GWAS methods to identify SNP effects. fastGWA and FarmCPU tend to identify SNPs with larger effects, particularly in regions with lower allele frequencies, suggesting that low-frequency alleles may play a crucial role in the genetic control of multiple phenotypes. In contrast, the BOLT-LMM method favors detecting SNPs with smaller effects, especially in the distribution of low-frequency alleles, indicating that low-frequency variants may have a stronger genetic contribution in some phenotypes. Additionally, the differences in MAF distribution further highlight the preference of different methods for identifying common versus low-frequency variants. This provides important insights into the genetic structure of these phenotypes, offering a deeper understanding of the underlying genetic architecture.

### 2.5. Consistency and Differences in the Detection of Important SNPs (p < 0.05) by Different GWAS Methods

**Figure 5:**
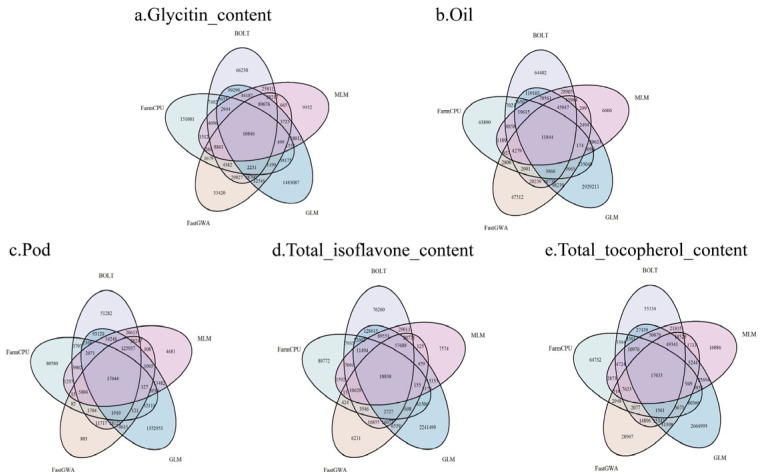
Venn Diagrams of important SNPs Identified by Different GWAS Methods Across Five Soybean Phenotypes. Each Venn diagram shows the number of important SNPs (*p* < 0.05) identified by BOLT, FarmCPU, fastGWA, GLM, and MLM for the respective phenotypes. The diagrams highlight the overlap and unique SNPs identified by each method, demonstrating the consistency and differences in SNP identification across methods.

Figure 5 shows the number of important SNPs (*p* < 0.05) identified by each GWAS method (BOLT-LMM, FarmCPU, fastGWA, GLM, MLM) across the five phenotypes, as well as the consistency and differences between them. The Venn diagram clearly illustrates the overlap and differences in the important SNPs detected by each method. For each phenotype, there were important differences in the number of important SNPs detected by the various methods. For the Glycitin content and Oil phenotypes, the number of important SNPs identified by FarmCPU and fastGWA were relatively similar, but there was minimal overlap with other methods, suggesting that these methods may focus on different genetic loci. Additionally, for the Total isoflavone content phenotype, the overlap between methods was more pronounced, with a larger intersection, indicating strong consistency among all methods in detecting the genetic variation for this trait. Overall, although there was some overlap in the important SNPs identified by the different methods, the number of important loci and the SNPs detected varied across phenotypes. This reflects differences in the sensitivity and preferences of each method when capturing genetic signals. These results highlight the complementary nature of different GWAS methods in phenotype analysis and also reveal that a single method may overlook certain genetic information. Therefore, combining multiple methods for GWAS analysis enables a more comprehensive capture of the genetic variation underlying phenotypes.

### 2.6. Genome Selection (GS) Analysis

**Figure 6:**
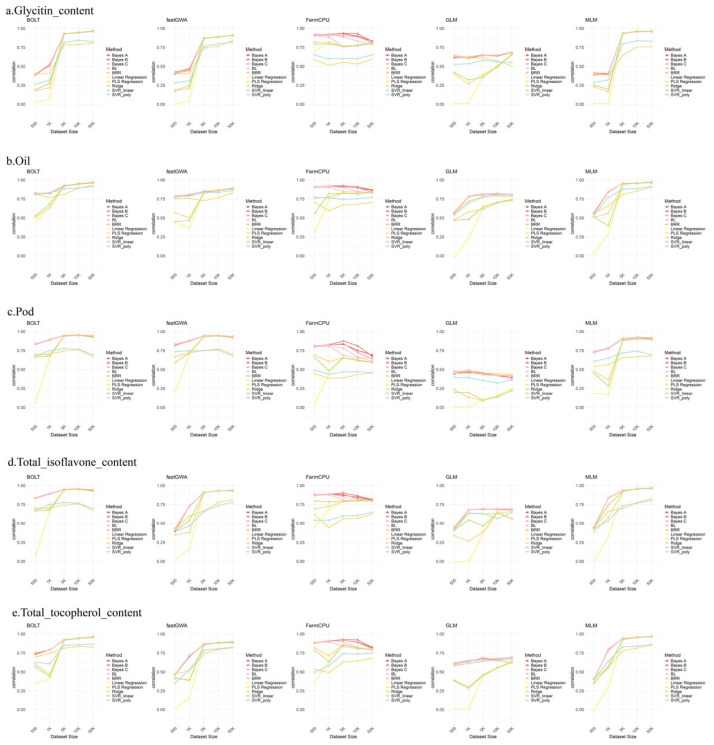
Comparison of Prediction Performance Across Different GS Models Using Varying SNP Densities. The plots show the prediction accuracy for each GS model across different SNP densities. Each panel compares the performance of multiple GS models, including BayesA, BayesB, BayesC, BL, BRR, SVR_poly, SVR_linear, Ridge, PLS_Regression, and Linear_Regression.

To assess the impact of varying SNP numbers on the performance of genome selection (GS) models, Figure 6 presents the results of prediction using different numbers of SNPs across five phenotypes. As the number of SNPs increased, the prediction accuracy of most models improved, particularly for BOLT, MLM, and fastGWA, indicating that these three models were more effective at capturing greater genetic variation. However, for FarmCPU and GLM, using around 5000 SNPs was sufficient to achieve high prediction accuracy, suggesting that the genetic information for these traits is concentrated in a smaller number of SNPs, and additional SNPs did not importantly enhance prediction capability. Furthermore, among all the models, GLM had the lowest prediction accuracy, likely due to its failure to correct for population structure, leading to false positives, which aligns with the GWAS results discussed earlier. The heritability estimates also support the notion that prediction accuracy importantly improved after GS analysis using the SNP markers identified by GWAS.

**Figure 7:**
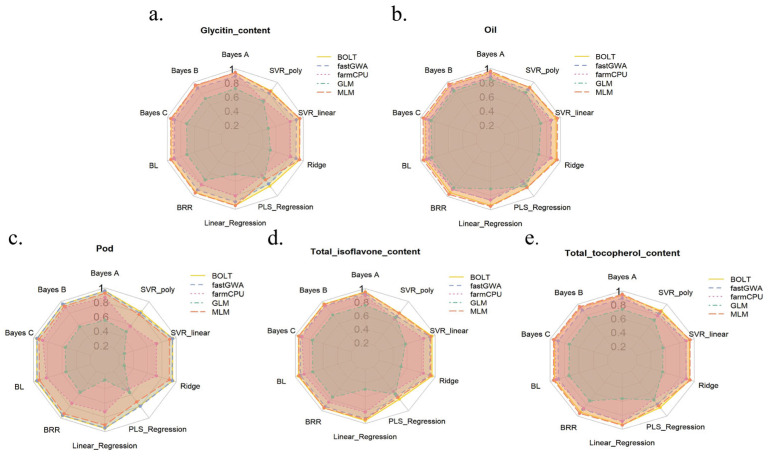
Radar Plots Comparing Prediction Performance of Different GS Models at 5 K SNP Density Across Soybean Phenotypes.. Each radar plot compares the prediction accuracy of various GS models, including BayesA, BayesB, BayesC, BL, BRR, SVR_poly, SVR_linear, Ridge, PLS_Regression, and Linear_Regression.

To further compare the results of different GS models, we selected 5 K density markers for additional analysis, as shown in Figure 7. We evaluated the prediction accuracy of various models, including BayesA, BayesB, BayesC, BL, BRR, SVR_poly, SVR_linear, Ridge, PLS_Regression, and Linear_Regression. The radar plots for each phenotype show the predictive ability of the different models. From the plots, it is evident that BayesA and BayesB performed the best for most phenotypes, particularly for Glycitin content, Oil, and Total tocopherol content, with their accuracy values approaching 1, indicating high predictive power. In contrast, SVR_poly and PLS_Regression performed poorly for certain phenotypes (such as Pod and Total isoflavone content), with relatively low accuracy values. This suggests that BayesA and BayesB models exhibit strong predictive power for genome selection in soybean, making them suitable for most phenotypes. On the other hand, models like SVR_poly and PLS_Regression may not provide sufficient accuracy in certain cases, highlighting the importance of considering phenotype characteristics and marker numbers when selecting an appropriate model.

## 3. Discussion

This study compared the performance of different statistical methods and models in prediction accuracy by conducting genome selection (GS) and genome-wide association studies (GWAS) on five important soybean phenotypes. Our analysis highlights the advantages and limitations of various methods, providing valuable insights for soybean genetic improvement and genome selection.

### 3.1. Comparison of GWAS Methods

In the GWAS analysis, we employed fastGWA, BOLT-LMM, FarmCPU, GLM, and MLM methods to analyze five phenotypes. The results indicated that although GLM identified the most important loci, it is a basic model that lacks correction for population structure, leading to a higher rate of false positives. This issue was also observed in the subsequent genome selection analysis. Both BOLT-LMM and FarmCPU played a important role in identifying key loci, which aligns with existing literature [21,22,23]. The application of FarmCPU in large-scale datasets has demonstrated its high efficiency in detecting both rare and common variants [24,25]. Furthermore, the differences in *p*-value distributions between methods underscore the importance of selecting the appropriate GWAS method. fastGWA and FarmCPU were relatively consistent in identifying important loci, while GLM and MLM showed more scattered *p*-values in some phenotypes and failed to detect critical genetic signals. This suggests that in practical applications, combining multiple methods for GWAS analysis provides a more comprehensive identification of genetic loci related to the phenotype.

### 3.2. Comparison of Genome Selection Models

For the comparison of genome selection (GS) models, we evaluated the prediction performance of BayesA, BayesB, BayesC, BL, BRR, SVR_poly, SVR_linear, Ridge, PLS_Regression, and Linear_Regression models [26,27]. The results indicated that BayesA and BayesB performed the best for most phenotypes, particularly for Glycitin content and Oil, where prediction accuracy reached approximately 0.95. This shows that these two models are effective in capturing the genetic information associated with phenotypic variation. This finding is consistent with previous studies, as BayesA and BayesB models are highly adaptable and capable of handling more complex genetic data, especially in cases involving polygenic effects.

In contrast, the SVR_poly and PLS_Regression models performed poorly for certain phenotypes, suggesting that these models may struggle to capture the nonlinear relationships in the data. Specifically, for the Pod and Total isoflavone content phenotypes, these models showed lower prediction accuracy, indicating that they may not be suitable for all types of genetic data. This finding further emphasizes the importance of selecting the appropriate GS model to improve prediction accuracy.

### 3.3. Impact of SNP Number and Models

Our analysis further revealed that the impact of the number of important SNPs on prediction accuracy varied importantly across different phenotypes. For most models, prediction accuracy improved with an increase in the number of SNPs, particularly when more than 5000 SNPs were used. In contrast, FarmCPU and GLM models achieved high prediction accuracy with approximately 5000 SNPs, suggesting that the genetic information for these phenotypes is primarily determined by a small number of SNPs. This indicates that different phenotypes may require varying numbers of SNPs for effective prediction, highlighting the need to optimize the selection of SNP numbers based on phenotype characteristics and genetic background in practical applications.

### 3.4. Role of Low-Frequency Alleles

In this study, the GWAS results and GS analysis for multiple phenotypes indicated that low-frequency alleles play an important role in the genetic control of soybean traits. Particularly for the Pod and Total tocopherol content phenotypes, the contribution of low-frequency SNPs was notably pronounced. The influence of low-frequency alleles may lie in their large effect sizes; however, their low frequency makes them difficult to capture using common SNPs [28,29,30]. This suggests that future research should focus more on the potential of low-frequency alleles in soybean genetic improvement, particularly in how these variants can be effectively utilized during the breeding process to enhance the expression of target traits.

Despite the valuable insights provided by this study, there are several limitations. First, the sample size and the quality of SNP data used may have impacted the predictive ability of some models, particularly in detecting low-frequency alleles. Additionally, this study focused on only five soybean phenotypes, and future research could expand the range of phenotypes to explore the genetic basis of other economically important traits, such as disease resistance and yield. Furthermore, with advancements in technology, new deep learning algorithms and high-throughput data analysis methods may offer enhanced predictive capabilities [7,31,32]. These methods warrant further exploration in future studies.

### 3.5. Limitations of the Study

Despite providing valuable results, this study has several limitations. First, the sample size and the quality of SNP data used may have affected the predictive accuracy of some models, particularly in the detection of low-frequency alleles. Additionally, this study focused on only five soybean traits, and future research could expand the phenotypic scope to explore the genetic basis of other economically important traits, such as disease resistance and yield.

Second, population diversity is an important factor influencing the generalizability of the study’s results. Our research samples were mainly derived from specific geographic regions and varieties, which may limit the broader applicability of the findings. If the genetic background and geographic distribution of the sample population are narrow, the results may only be applicable to specific environments or varieties. Therefore, future studies should aim to expand the diversity of the sample, including more samples from different regions and varieties, to enhance the generalizability of the conclusions.

With the advancement of technology, new machine learning algorithms and high-throughput data analysis methods may offer improved predictive capabilities, and these methods warrant further exploration in future research.

## 4. Materials and Methods

### 4.1. Data Sources

The genotype data used in this study were derived from a re-sequencing project conducted by Caiying Zhang et al. [33] in 2022, which included 547 soybean germplasm samples from China and the United States, comprising 61 local varieties and 486 improved cultivars. The research team first assembled a high-quality genome for the modern soybean cultivar NDD2 (Nongda 2) and used it as a reference genome for SNP (single nucleotide polymorphism) detection. Phenotypic data were obtained from these 547 re-sequenced germplasms, which were grown in 10 different environments in 2019, 2020, and 2021. After filtering both phenotypic and genotypic information, a total of 464 samples were selected for this study, focusing on five phenotypes: Glycitin content, Oil, Pod, Total isoflavone content, and Total tocopherol content.

### 4.2. Estimation of Heritability

To better understand the genetic mechanisms underlying soybean traits and reveal the respective contributions of genetic and environmental factors, this study assessed the heritability of five soybean traits. Heritability was estimated using the fastGWA-REML method in GCTA software, and the significance of the heritability estimates was tested using *p*-values [34].

### 4.3. GWAS Methods

To analyze the association between soybean traits and genotypes, multiple GWAS methods were employed, including the fastGWA method in GCTA [35], the BOLT-LMM method in BOLT [11], and the FarmCPU, GLM, and MLM methods from the rMVP package in R [36]. The fastGWA method in GCTA utilizes a linear mixed model (LMM) to effectively control for population structure and individual relatedness [12,37]. Specifically, the genetic relationship matrix (kinship matrix) derived from genotype data is used to model genetic correlations between individuals, reducing the confounding effect of population structure on the analysis results. The BOLT-LMM algorithm also uses an LMM to calculate association statistics between phenotypes and genotypes. The default settings of this algorithm employ a Bayesian mixture normal distribution prior to modeling the random effects of SNPs unrelated to the SNPs being tested. The FarmCPU method combines both fixed and random effect models, performing genetic marker selection in two stages to minimize the interference of population structure. By alternating between fixed and random effect models, this approach enhances both the sensitivity and accuracy of the analysis. The GLM method is a basic model commonly used in GWAS, which includes fixed effects (e.g., environmental variables and covariates) in the model. The MLM model incorporates both fixed and random effects to improve result accuracy and reduce false positives. This method is particularly suitable for handling complex genetic data, as it accounts for both genetic relationships (kinship) and population structure.

Each of the five traits was analyzed independently using these five models, and the results were visualized using CMplot (R package(Version 4.4)) to generate Manhattan and QQ plots [38]. To compare the differences between models, the distribution of β effect sizes and MAF values, as well as the number of important markers, were calculated and analyzed. MAF (Minor Allele Frequency) refers to the frequency of the least common allele at a given SNP. The calculation formula is as follows:

$$MAF=min\left(\frac{N_{allele1}}{N_{total}},\frac{N_{allele2}}{N_{total}}\right)$$

Here, $N_{allele1}$ represent the counts of the two alleles, and $N_{allele2}$ is the total number of samples. The lower the MAF value, the more likely the SNP is a low-frequency allele, whereas a higher MAF indicates a high-frequency allele. β (regression coefficient) represents the effect size of each SNP on the target trait, typically calculated through regression models. In GWAS analysis, β is estimated using a linear regression model, as shown in the formula below:

$$Y=\beta_{0}+\beta_{1}X+\epsilon$$

where Y is the phenotypic value of the target trait; X is the genotype of the SNP (typically encoded as 0, 1, or 2, representing different allele combinations); $\beta_{0}$ is the intercept; $\beta_{1}$ is the regression coefficient, representing the effect size of the SNP on the target trait; ϵ is the error term. The $\beta_{1}$ value calculated through regression analysis indicates the strength of the SNP’s effect on the trait, with positive values representing a positive effect and negative values indicating a negative effect.

### 4.4. Division of Different Densities

In this study, the association between each marker and trait was evaluated using *p*-values derived from GWAS. To explore the impact of different marker densities on subsequent GS, markers were sorted by *p*-value in ascending order, with smaller *p*-values indicating higher significance. For each method and trait, markers were classified into five density gradients: 500, 1 K, 5 K, 10 K, and 50 K. Subsequently, the marker information was extracted using bcftools, resulting in a total of 125 datasets (5 phenotypes × 5 models × 5 marker densities) for the subsequent GS analysis [39]. In GWAS analysis, the *p*-value is typically calculated using the following formula:

$$P=P(\mathrm{observed}\;\mathrm{effect}\;\geq \;\mathrm{expected}\;\mathrm{effect}|H_{0}\mathrm{is}\mathrm{true})$$

That is, under the null hypothesis ($H_{0}$, which assumes no association between the marker and the trait), the *p*-value is calculated as the probability of observing the effect (typically the regression coefficient or difference) given that the null hypothesis is true. If the *p*-value is small (typically less than 0.05), the null hypothesis is rejected, indicating an important association between the marker and the trait.

### 4.5. GS Analysis

After obtaining 125 datasets from the GWAS analysis, GS was performed using 10 models (5 Bayesian models + 5 machine learning models). Each model underwent 5-fold cross-validation, repeated 10 times (5 × 10), resulting in a total of 50 experiments. The BGLR package in R was used for the Bayesian models, including BayesA, BayesB, BayesC, BL, and BRR, while the sklearn library in Python(Version 3.8) was used for the machine learning models, which included Linear Regression, PLS Regression, Ridge, SVR_linear, and SVR_poly [40,41,42]. To ensure the stability of the results, the average of the 50 experiments was selected for comparison. The Pearson correlation coefficient (r) was calculated to assess the linear correlation between the predicted results of different models and the actual phenotypic values. The formula for calculating the Pearson correlation coefficient is as follows:

$$r=\frac{\sum \left(X_{i}-\overset{‾}{X}\right)\left(Y_{i}-\overset{‾}{Y}\right)}{\sqrt{\sum {\left(X_{i}-\overset{‾}{X}\right)}^{2}\sum {\left(Y_{i}-\overset{‾}{Y}\right)}^{2}}}$$

where $X_{i}$ and $Y_{i}$ represent the predicted and actual values, respectively, and $\bar{X}$ and $\bar{Y}$ represent the mean of the predicted and actual values, respectively. Finally, ggplot2 (R package(Version 4.4)) was used for visualization to display the predictive performance of different models [43].

## 5. Conclusions

This study explored the application of different GWAS-GS models in soybean genetic improvement by conducting genome-wide association studies (GWAS) on five important soybean phenotypes (Glycitin content, Oil, Pod, Total isoflavone content, Total tocopherol content), followed by genome selection (GS) model evaluation. The results demonstrate that the use of SNP markers identified through GWAS importantly improved prediction accuracy, with some combinations reaching a prediction accuracy of approximately 0.95. Among the GWAS models, FarmCPU and BOLT performed the best, while in the GS models, Bayesian models were more stable compared to machine learning models. Through comparisons of different methods and SNP numbers, we found considerable diversity in the genetic control of phenotypes. Some phenotypes could be effectively predicted with a smaller number of SNPs, while others required more SNPs to enhance prediction accuracy. The varying performance of different GS models across phenotypes further underscores the importance of model selection. Overall, this study provides new insights into soybean genome selection, highlights the importance of low-frequency alleles, and offers guidance for future breeding efforts by recommending appropriate GS models. By combining various analytical methods and selecting the appropriate number of SNPs, more precise genetic improvement of soybean can be achieved, providing scientific support for enhancing crop yield and quality.

## Figures and Tables

**Table 1:** Heritability Estimates for Five Soybean Phenotypes. The table presents the heritability (h^2^), *p*-values for heritability, genetic variance (V_g_), and environmental variance (V_e_) for each phenotype, estimated using the fastGWA_REML method. The values indicate the proportion of phenotypic variation attributed to genetic factors and the significance of genetic influence on the traits.

Trait	Heritability	Pval	V_g_	V_e_
Glycitin_content	0.58337	2.70049 × 10^−12^	1705.95 ± 243.969	1218.31 ± 125.792
Oil	0.62777	8.4279 × 10^−20^	1.18832 ± 0.13047	0.70460 ± 0.06681
Pod	0.17354	0.00136	34.0584 ± 10.6366	162.193 ± 15.3207
Total_isoflavone_content	0.46592	9.9697 × 10^−9^	11590 ± 20,222.9	132,855 ± 13,745.6
Total_tocopherol_content	0.58590	7.36219 × 10^−14^	668.987 ± 89.4219	472.818 ± 45.6027

**Table 2:** Computation time for each GWAS model on a single trait. The times represent the average running time for each model, with all models evaluated on the same trait. The BOLT and fastGWA models showed shorter computation times compared to other methods.

**Model**	**Time**
BOLT	1 min 58 s
fastGWA	1 min 12 s
FarmCPU	5 min 46 s
GLM	4 min 49 s
MLM	5 min 40 s

## Data Availability

The original contributions presented in this study are included in the article. Further inquiries can be directed to the corresponding authors.
